# Determination of glucose exchange rates and permeability of erythrocyte membrane in preeclampsia and subsequent oxidative stress-related protein damage using dynamic-^19^F-NMR

**DOI:** 10.1007/s10858-017-0092-y

**Published:** 2017-02-21

**Authors:** Elizabeth Dickinson, John R. P. Arnold, Julie Fisher

**Affiliations:** 1 Department of Chemistry, University of York, Heslington, York, UK; 2 Selby College, Abbot’s Road, Selby, North Yorkshire YO8 8AT UK; 3 School of Chemistry, University of Leeds, Leeds, UK

**Keywords:** NMR, Dynamic, Exchange, Membrane, Preeclampsia, Oxidative stress

## Abstract

The cause of the pregnancy condition preeclampsia (PE) is thought to be endothelial dysfunction caused by oxidative stress. As abnormal glucose tolerance has also been associated with PE, we use a fluorinated-mimic of this metabolite to establish whether any oxidative damage to lipids and proteins in the erythrocyte membrane has increased cell membrane permeability. Data were acquired using ^19^F Dynamic-NMR (DNMR) to measure exchange of 3-fluoro-3-deoxyglucose (3-FDG) across the membrane of erythrocytes from 10 pregnant women (5 healthy control women, and 5 from women suffering from PE). Magnetisation transfer was measured using the 1D selective inversion and 2D EXSY pulse sequences, over a range of time delays. Integrated intensities from these experiments were used in matrix diagonalisation to estimate the values of the rate constants of exchange and membrane permeability. No significant differences were observed for the rate of exchange of 3-FDG and membrane permeability between healthy pregnant women and those suffering from PE, leading us to conclude that no oxidative damage had occurred at this carrier-protein site in the membrane.

**Electronic supplementary material:**

The online version of this article (doi:10.1007/s10858-017-0092-y) contains supplementary material, which is available to authorized users.

## Introduction

Preeclampsia (PE) is a human-specific hypertensive disorder of pregnancy which manifests itself after 20 weeks of gestation, developing in almost 10% of pregnancies (El Hassan et al. 2015; Kumru et al. 2006). The cause of PE is still not fully understood, though it is a major cause of maternal and foetal morbidity and mortality. PE affects the mother by vascular endothelial dysfunction and causes intrauterine growth restriction of the foetus (Hubel 1999; Poston et al. 2006). Delivery of the baby is the only method of reversing the syndrome.

Numerous etiologies have been asscociated with PE, though the attack of reactive oxygen species (ROS), and oxidative stress, is thought to be the most important factor contributing to the pathogenesis of PE, causing uncontrolled lipid peroxidation, protein modification and changes to cell membrane structure (Raijmakers et al. 2008; Ethordevic et al. 2008; Adiga et al. 2007; Mohan and Venkataramana 2007; Shoji et al. 2008; Shoji and Koletzko 2007). It has been suggested that polyunsaturated fatty acids are attacked by ROS and converted into lipid hydroperoxides as the initial factor leading to vascular endothelial dysfunction in PE (Howlander et al. 2007; Kaur et al. 2008; Hubel et al. 1989; Davidge et al. 1992; Mehendale et al. 2008; Patil et al. 2007).

Products of lipid peroxidation have the ability to cause further oxidative damage by attacking proteins present in the cells and tissue, which in turn causes lysis of erythrocytes (Salvi et al. 2001; Negre-Salvayre et al. 2008; Davies 1987; Esterbaur et al. 1991). Oxidative damage to membrane proteins can also occur by direct free radical attack—such protein modifications can cause structural changes and may also result in detrimental changes in their function, affecting activity of enzymes, receptors or membrane transporters (Roche et al. 2008; Jones 2008; Stadtman 1993).

Abnormal glucose tolerance has also been implicated as a risk factor for PE (Joffe et al. 1998; Parra-Cordero et al. 2014). Glucose enters and leaves the red blood cell by passive transport via an intrinsic protein, GLUT1, so that the facilitated diffusion of this species in and out of erythrocytes is in dynamic equilibrium (May 1998; Gabel et al. 1997; O’Connell et al. 1994; Potts et al. 1990; Potts and Kuchel 1992; London and Gabel 1995). However, any damage to membrane proteins or lipids due to oxidative stress may cause changes in their conformation, which could affect the rate at which the diffusion of species across the membrane occurs. We have demonstrated previously that NMR is capable of identifying metabolomic differences in the blood of healthy pregnant women and those suffering from PE (Turner et al. 2007, 2008, 2009). We therefore used NMR spectroscopy to investigate the hypothesis that a difference in glucose exchange rate and cell membrane permeability will be observed between healthy pregnant women and those suffering from PE.

NMR exchange experiments allow processes to be investigated under dynamic equilibrium (Perrin and Dwyer 1999; Perrin and Engler 1990; Perrin and Gipe 1984; Robinson et al. 1985). This is achieved by monitoring the transfer of longitudinal magnetisation during a delay in the NMR pulse sequence applied (Grassi et al. 1986; McConnell 1958; Forsén and Hoffman 1963; Muhandiram and McClung 1987). In the application of dynamic NMR (D-NMR) to the study of exchange in erythrocytes from pregnant women, both one dimensional and two-dimensional spectra have been recorded to detect exchange pathways and determine rates of exchange and relaxation of species. By applying both experiments, the results obtained for each can be compared and confirmed, to ensure that the estimates of the values of the rate constants obtained are reliable. As demonstrated previously, 2D methods provide a qualitative map of the exchange process and are tolerant to small differences in chemical shift between the peaks involved in exchange (Johnston et al. 1986; Macura and Ernst 1980); the 1D methods provide faster data acquisition and analysis, especially for two-site systems, as long as the exchange network is known (Perrin and Engler 1990; Robinson et al. 1985; Engler et al. 1988). The integrated intensities obtained from the 1D and 2D experiments can then be used to estimate the values of the first order rate constants of exchange, and establish if oxidative damage has indeed compromised the erythrocyte membrane (Gabel et al. 1997; Perrin and Dwyer 1999; Perrin and Gipe 1984). Whilst the processes of exchange and the nuclear Overhauser effect are different, both rely on the transfer of longitudinal magnetisation, which is why the same pulse sequence can be applied.

Several one-dimensional experiments exist based on the NOESY pulse sequence, which can be employed to measure the transfer of magnetisation (Robinson et al. 1985; Bellon et al. 1987; Engler et al. 1988; Bulliman et al. 1989; Perrin and Engler 1990). Whilst the “Overdetermined” 1D EXSY pulse sequence of Bulliman et al. (1989) has also been used to study erythrocytes (Potts and Kuchel 1992), the more complex matrix diagonalisation methods are different to those employed in most other exchange applications. Selective inversion was the 1D method of choice in this investigation (Robinson et al. 1985). The exchange of species monitored by 2D spectroscopy was initially documented by Jeener et al. (1979), and has become invaluable in establishing the mechanisms of exchange (Perrin and Gipe 1984; Meier and Ernst 1979; Macura and Ernst 1980; Bremer et al. 1984; Johnston et al. 1986; Montelione and Wagner 1989). The principle is similar, as expected, to that for the selective inversion experiment and so should give comparable results for the elements of the rate matrix. Exchange occurs during the mixing time and in the 2D EXSY four peaks will be produced in the two-site case of cellular exchange i.e. two cross peaks and two diagonal peaks (Gabel et al. 1997; O’Connell et al. 1994; Kirk and Kuchel 1985; Potts et al. 1989). The volumes of all these peaks can then be used in matrix diagonalisation to estimate the values of the exchange rate and relaxation rate constants. A full explanation of the matrix diagonalization method has been included in the Appendix for completeness, as the procedure is not used nor fully described very often in the literature.

By estimating the values of the rate constants of cellular exchange, the measurement of the permeability of the erythrocyte membrane is possible. This will give information on the condition of the membrane with a higher permeability in PE providing an indication of oxidative stress or attack and compromise of the lipid bilayer by ROS.

The inward permeability is calculated from the inward rate constant, previously determined from the NMR data:

1 $${{P}_{1}}=\frac{{{V}_{e}}}{A}{{k}_{1}}=\frac{MCV\left( 1-Ht \right)}{{{A}_{cell}}\left( Ht \right)}{{k}_{1}}$$

here *Ht* is the haematocrit (or red blood cell count); *V*_*e*_ is the extracellular volume (mL), calculated as *V*_*o*_(1-*Ht*), where *V*_*o*_ is the NMR sample volume; *A* is the total surface area of the cells, calculated from (*A*_*cell*_ *Ht*)/MCV, where *A*_*cell*_ = 1.43 × 10^− 6^ cm^2^ and MCV (mean cell volume) = 85 fL for erythrocytes in isotonic solution; *k*_1_ = influx rate constant (Raftos et al. 1990; O’Connell et al. 1994; London and Gabel 1995; Chapman and Kuchel 1990).

Similarly, the outward permeability can be calculated using the efflux rate constant:

2 $${{P}_{-1}}=\frac{{{V}_{i}}}{A}{{k}_{-1}}=\frac{MCV{{f}_{w}}}{{{A}_{cell}}}{{k}_{-1}}$$

where *f*_*w*_ is the fraction of red cell volume which is accessible to solutes, and *k*_− 1_ is the efflux rate constant (Raftos et al. 1990; O’Connell et al. 1994; London and Gabel 1995). It is clear from Eq. (2) that the outward permeability is independent of haematocrit.

## Materials and methods

### Patient selection and sample preparation

Women chosen for this part of the study were all beyond 20 weeks of gestation and were attending The Leeds Teaching Hospitals NHS Trust, Leeds, UK. The women were of any ethnicity and were not all in their first pregnancy. The PE group exhibited fully established PE, diagnosed according to the criteria of American College of Obstetrics and Gynecologists (ACOG) i.e., a rise in blood pressure after 20 weeks gestation to >140/90 mm Hg on two or more occasions 6 h apart in a previously normotensive woman, combined with proteinuria (Davey and MacGillivray 1988). Proteinuria was defined as protein dipstick >1 + on two or more midstream urine samples, or a 24 h urine excretion of >0.3 g protein, in the absence of a urinary tract infection (Harsem et al. 2006). Healthy control women were generally from later in pregnancy, i.e. >30 weeks, to ensure that they remained healthy controls and did not develop PE weeks after sample collection. Venous blood was collected in heparinized (lithium salt) anticoagulant tubes. All fresh whole blood was centrifuged for 6 min at 3000 g and 4 °C, before removing and discarding the plasma and buffy coat. The same conditions were used in all subsequent washings of erythrocytes. For the transport of 3FDG, a saline buffer solution was prepared containing 132 mM NaCl, 15 mM Tris-HEPES (pH 7.4), 5 mM ascorbic acid and 10 mM 3FDG (O’Connell et al. 1994; Pallotta et al. 2014). Erythrocytes (still in the anticoagulant tube) were washed with the saline buffer solution in D_2_O containing the fluorinated glucose, using approximately three times the volume of the RBCs. The tube was inverted three times to mix the solution and RBCs before repeating centrifugation, and removing and discarding the wash solution. This washing procedure was repeated three times. After washing, carbon monoxide gas was bubbled through the cells for approximately 30 s with gentle stirring to remove deoxyhaemoglobin and paramagnetic O_2_ from the sample (O’Connell et al. 1994). Finally, the haematocrit of the sample was measured in duplicate using heparinised capillary tubes, and spun at approximately 1300 g for 5 min using a Haematospin 1300 (Hawsley, Lancing, Sussex, UK). It was assumed that 0.717 of the intracellular volume was accessible to the 3FG molecules (Potts and Kuchel 1992).

The RBCs were incubated at 37 °C for 1 h, before transferring 700 μl of the RBCs/glucose solution to an NMR tube for analysis.

### 1D ^19^F spectra fluorinated glucose in D_2_O

The 1D ^19^F-NMR FID of 5 mM 3-fluoro-3-deoxyglucose (3FDG) in D_2_O was acquired at 470.34 MHz and at 37 °C into 65,536 data points, using a relaxation delay of 5 s, a pulse duration of 10 µs, over 4 transients, at a temperature of 20 °C. An exponential line broadening of 1 Hz was applied to the FID, prior to zero filling to 131,072 points, followed by Fourier transformation. Resultant spectra were phased and baseline corrected using Vnmr 6.1 C (Varian Inc., Palo Alto, California, USA).

### 1D ^19^F spectra of RBCs and fluorinated glucose, with and without proton decoupling

Two one dimensional spectra were acquired of the RBCs and fluorinated glucose at 470.34 MHz and at 37°C, where broadband proton decoupling was applied in the second experiment. This allowed the ^19^F intracellular and extracellular resonances to be resolved without the complication of the geminal ^1^H-^19^F coupling (Gabel et al. 1997). For both experiments, an interpulse relaxation delay of 8 s was used, a delay which was longer than 5*T*_1_ (O’Connell et al. 1994). The ^19^F 90° pulse duration was determined for each new sample though was often 17 µs. The coupling constant measured in the first 1D spectrum was used in the calibration of the ^1^H 90° pulse duration for the proton decoupling in the second experiment, during which WALTZ decoupling was applied for the duration of the pulse and acquisition. 128 transients were collected into 16,384 data points for each spectrum, with a spectral width of 10,000 Hz. An exponential line broadening of 1 Hz was applied to each of the FIDs, prior to zero filling to 32,768 points, followed by Fourier transformation. Resultant spectra were phased, baseline corrected and integrated using Vnmr 6.1 C (Varian Inc., Palo Alto, California, USA). Manual integration was repeated and the mean average taken to minimise errors.

### Selective inversion

One dimensional ^19^F magnetization transfer experiments were performed on RBCs and fluorinated glucose at 470.34 MHz and at 37°C using the selective inversion method (O’Connell et al. 1994; Gabel et al. 1997; Robinson et al. 1985). Two series of experiments were performed for each anomer, using the 1D NOESY pulse sequence [RD–90˚_*x*_–*t*_1_–90˚_*x*_–*t*_m_–90˚_*x*_–acq], where either the intracellular or the extracellular peak was selectively inverted by setting the transmitter offset to the frequency of the resonance to be inverted. RD represents a relaxation delay of 8 s, a delay which was longer than 5*T*_1_ (O’Connell et al. 1994). The delay *t*_1_ = 1/(2|*ν*_i_-*ν*_e_|), where *ν*_i_ and *ν*_e_ are the frequencies of the intracellular and extracellular peaks respectively. The mixing time *t*_m_ was arrayed at delays of 0.001 (nominal zero), 0.05, 0.075, 0.10, 0.15, 0.30 and 0.45 s. After calibration of the ^1^H 90° pulse duration, broadband proton decoupling was applied using WALTZ decoupling during the final pulse and acquisition. The ^19^F 90° pulse duration used in the initial one dimensional experiments was applied (often 17 µs). 128 transients were collected into 16,384 data points for each spectrum, with a spectral width of 10,000 Hz. Again, exponential line broadening of 1 Hz was applied to each of the FIDs, prior to zero filling to 32,768 points, followed by Fourier transformation. Resultant spectra were phased, baseline corrected and integrated using Vnmr 6.1 C (Varian Inc., Palo Alto, California, USA). Manual integration was repeated and the mean average taken to minimise errors.

### 2D EXSY

Four two dimensional magnetization transfer experiments were performed on the RBC and fluorinated glucose samples at 470.34 MHz and at 37°C, using the broadband proton decoupled 2D NOESY pulse sequence [RD–90˚_*x*_–*t*_1_–90˚_*x*_–*t*_m_–90˚_*x*_–acq] (Gabel et al. 1997; Macura and Ernst 1980; Johnston et al. 1986). Each experiment had a different mixing time, *t*_m_, of either 0, 200, 400 or 600 ms. In all four experiments, 8 transients were collected into 4,096 data points in the directly detected dimension and 64 points in the second dimension, with a spectral width of 10,000 Hz. The same relaxation delay as in the 1D experiments was used (8 s), as well as the same previously calibrated ^19^F and ^1^H (for decoupling) pulse widths. Proton decoupling was provided in the directly detected dimension by application of WALTZ decoupling during the final pulse and acquisition. An exponential line broadening of 2 Hz was applied in both dimensions to each FID (O’Connell et al. 1994), prior to zero filling the second dimension to 2048 points, followed by Fourier transformation. Resultant spectra were phased and baseline corrected using Vnmr 6.1C (Varian Inc., Palo Alto, California, USA). Each spectrum was integrated using Lorentzian Fitting mode in the software Sparky 3.114 (T. D. Goddard and D. G. Kneller, SPARKY 3, University of California, San Francisco, USA), where peaks within a contour boundary were grouped, and where the data that were used were above the lowest contour.

### Matrix diagonalisation of integrated intensities

Integrated peak data from the 1D and 2D magnetization transfer experiments were analysed by matrix diagonalization using the software Maple 11 (Maplesoft, Waterloo Maple Inc, Waterloo, Ontario, Canada). Plots of the linearised matrix data were produced in Microsoft Excel (Microsoft Corporation, Redmond, WA USA), where the gradients of the lines in the plots were equal to elements of the relaxation matrix.

### Statistical analysis

After tests of normality had been performed, comparison of mean values (of integrated peaks, or rates of exchange) were performed, between the PE group and control group, using the *t* test or Mann–Whitney test in SPSS 13.0 software (SPSS Inc., Chicago, Illinois, USA). All *p* values were adjusted for multiple comparisons using false discovery rate in the software R 2.4.1 (R Foundation for Statistical Computing, Vienna, Austria), and values of <0.05 were regarded as statistically significant.

## Results

**Fig. 1:**
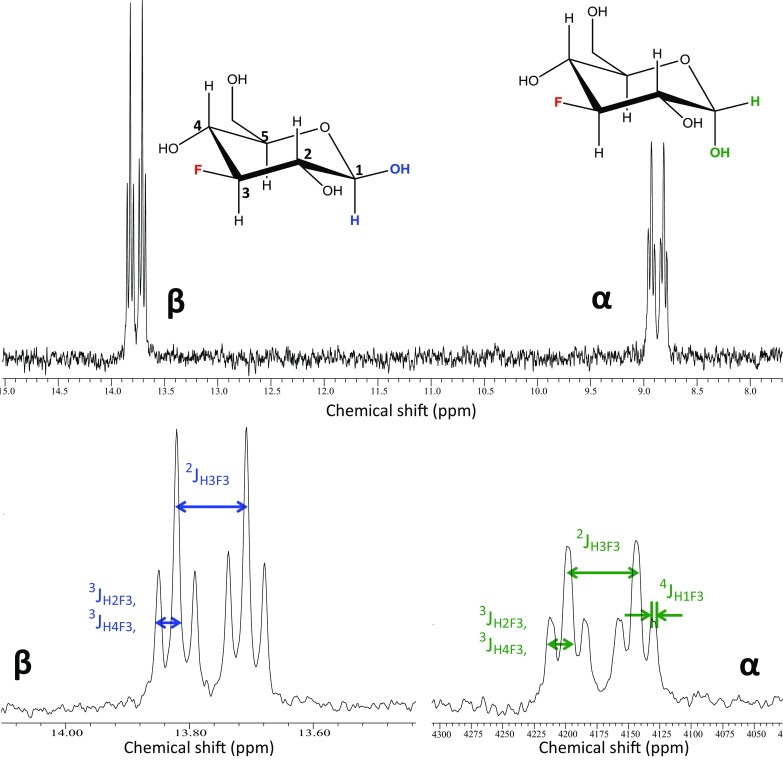
^19^F NMR spectrum and structure of 3FDG in D_2_O, at 470.34 MHz and at 37°C

**Fig. 2:**
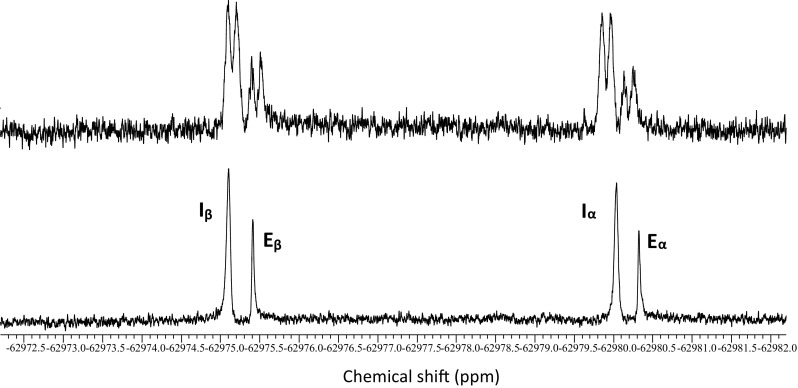
Spectra of erythrocytes washed with 3FDG solution (buffered with Tris-HEPES) where the bottom spectrum is broadband proton decoupled; I is intracellular 3-FDG and E is extracellular 3FDG (Ht = 79%)

**Fig. 3:**
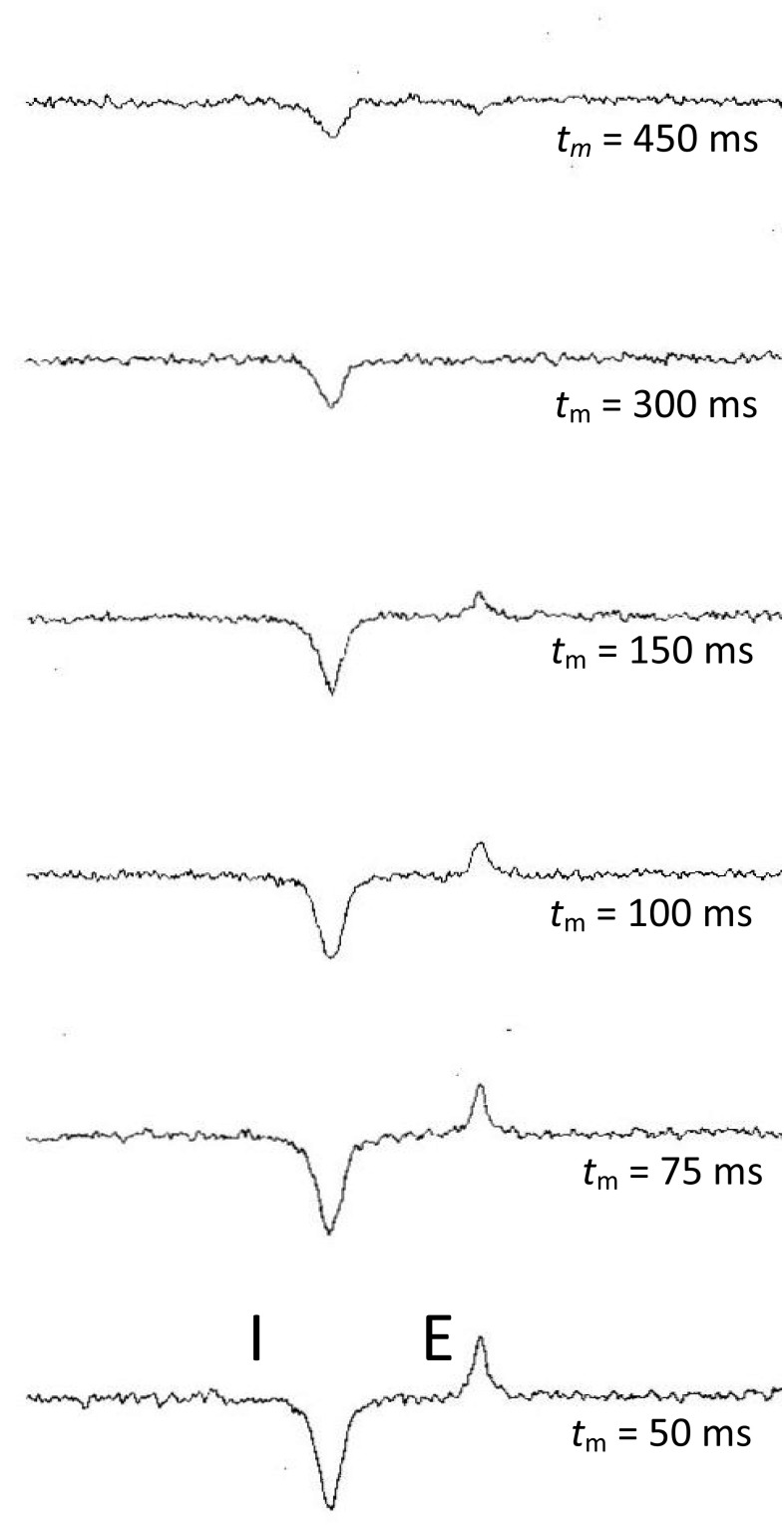
Expansions of the signals for the β-anomer of 3FDG, in exchange across the erythrocyte membrane, from the ^19^F-NMR 1D Selective Inversion spectra, acquired over a range of mixing times, *t*_m_ (Ht = 79%)

**Fig. 4:**
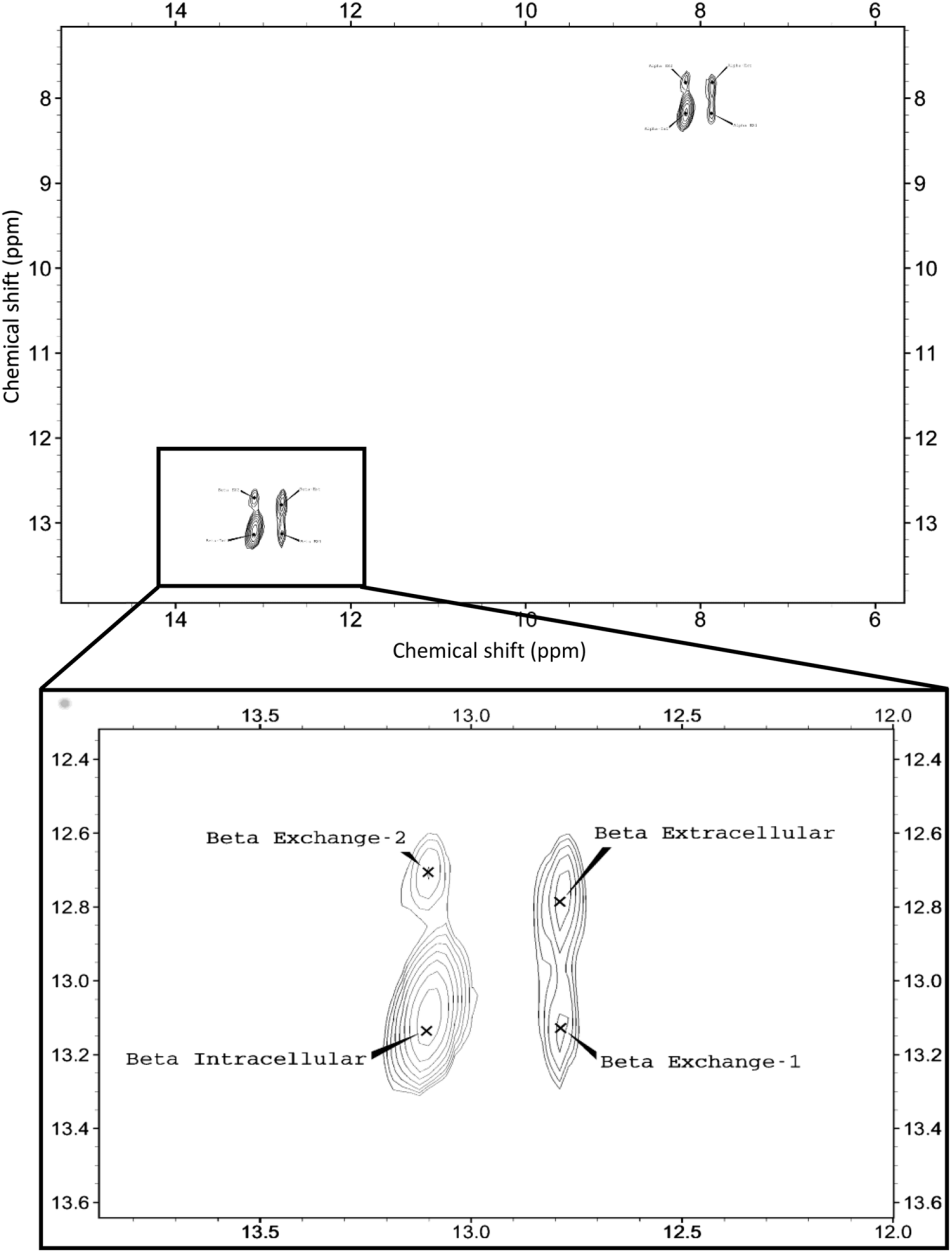
2D EXSY ^19^F-NMR spectrum, with expansion of β-anomer peaks, of erythrocytes washed in 3FDG solution; no cross peaks between the anomers shows that mutarotation is slow on the timescale of the experiment (Ht = 79%)

**Fig. 5:**
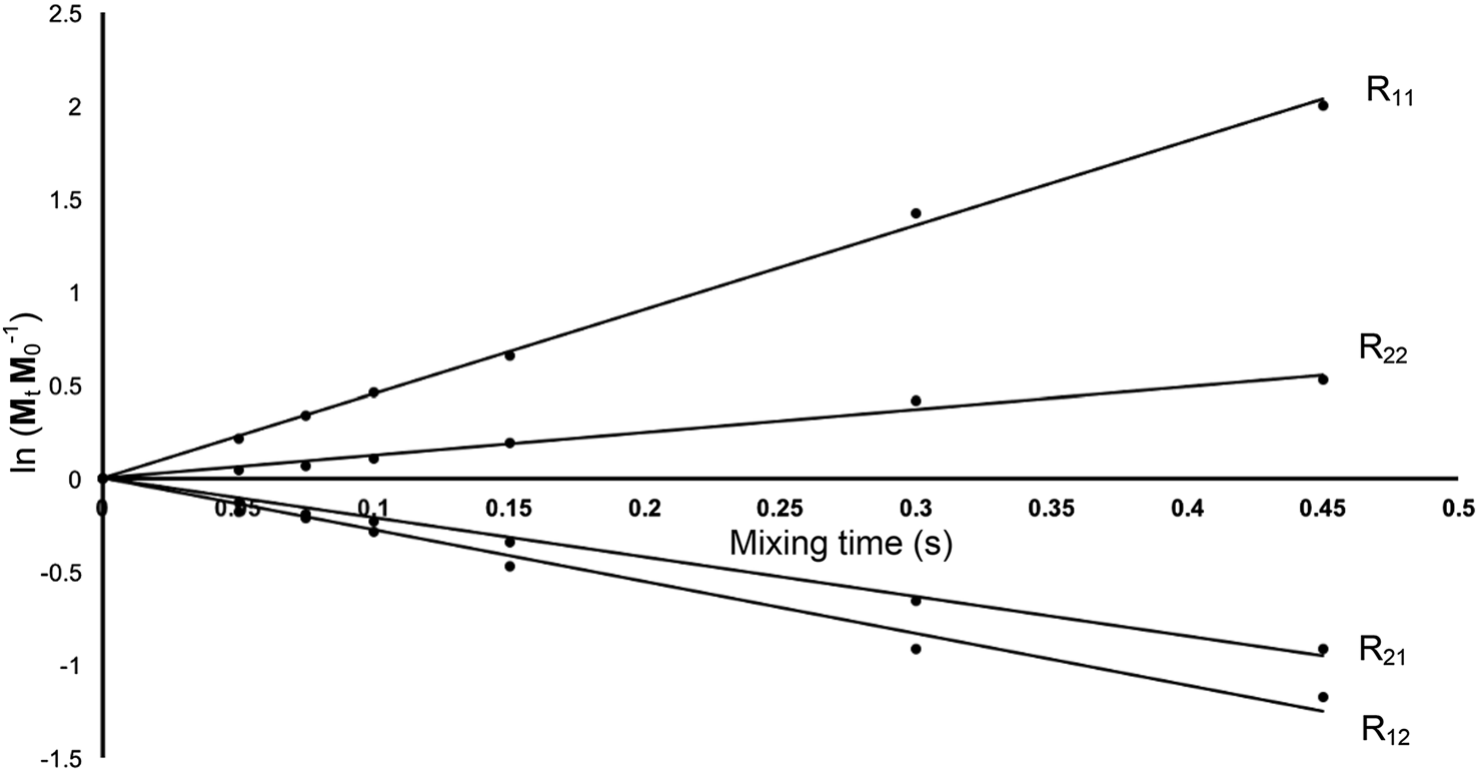
Plot of the linearised data from the exchange equation as function of mixing time (s), giving the elements of the rate matrix, R, for a control blood sample. R_11_ ($\frac{1}{{{T}_{{{1}_{i}}}}}+{{k}_{io}}$) = 4.52 s^− 1^ (*r* ^2^ = 1.00); R_12_ (*k*_oi_) = 2.78 s^− 1^ (*r* ^2^ = 0.98); R_21_(*k*_io_) = 2.11 s^− 1^ (*r* ^2^ = 0.99); and R_22_ ($\frac{1}{{{T}_{{{1}_{o}}}}}+{{k}_{oi}}$) = 1.22 s^− 1^ (*r* ^2^ = 0.98)

1D ^19^F spectra of 3FDG and washed erythrocytes are shown in Figs. 1 and 2 respectively, as well as examples of the 1D Selective Inversion (Fig. 3) and 2D EXSY (Fig. 4) magnetisation transfer experiments. Figure 3 shows the 2D EXSY spectrum of red blood cells washed with exchanging 3FDG. It is clear that mutarotation between anomers is too slow to occur on the timescale of the experiment, as no chemical exchange peaks are present between the β- and α-anomer. This allowed a simplification of the matrix diagonalisation methods; each anomer was treated as a separate probe, therefore producing 2, 2 × 2 rate matrices, rather than 1, 4 × 4 matrix (O’Connell et al. 1994; Gabel et al. 1997; Macura and Ernst 1980; Johnston et al. 1986). An example of a plot of the linearised data from the exchange equation is shown in Fig. 5. The mean average elements of the rate matrix, estimated from the magnetisation transfer experiments, and the calculated permeabilities for each anomer are shown in Table 1.

**Table 1:** Results of both 1D and 2D magnetisation transfer experiments on 3FDG exchange (errors are one standard deviation)

Anomer	Element of rate matrix and permeabilities		1D Selective inversion			2D EXSY		
			PE, n = 5	CONTROL, n = 5	*p*-value	PE, n = 5	CONTROL, n = 5	*p*-value
β	$\frac{1}{{T_{{1_{i} }} }} + k_{{io}} ~(s^{{ - 1}} )$	R_11_	4.67 ± 0.85	4.64 ± 0.82	0.841	1.85 ± 0.55	1.81 ± 0.10	0.730
	*k*_oi_ (s^−1^)	R_12_	3.06 ± 0.40	3.23 ± 0.52	0.548	3.32 ± 0.49	3.61 ± 0.62	0.841
	***k***_**io**_ (s^− 1^)	**R**_**21**_	**2.13** ± **0.07**	**2.14** ± **0.14**	**0.841**	**2.40** ± **0.31**	**2.12** ± **0.23**	**0.286**
	$\frac{1}{{T_{{1_{o} }} }} + k_{{oi}} ~(s^{{ - 1}} )$	R_22_	1.50 ± 0.18	1.40 ± 0.16	0.421	1.41 ± 0.59	1.03 ± 0.06	0.190
	Inward permeability (cm s^− 1^)		3.85 ± 0.56 × 10^− 5^	3.73 ± 0.51 × 10^− 5^	1.000	4.15 ± 0.54 × 10^− 5^	4.16 ± 0.68 × 10^− 5^	1.000
	Outward permeability (cm s^− 1^)		9.06 ± 0.31 × 10^− 5^	9.13 ± 0.59 × 10^− 5^	0.841	1.01 ± 0.13 × 10^− 4^	9.25 ± 0.94 × 10^− 5^	0.286
	$\frac{{\text{P}}_{\text{oi}}}{{\text{P}}_{\text{io}}}$		0.42 ± 0.05	0.41 ± 0.05	0.421	0.42 ± 0.05	0.46 ± 0.12	0.556
α	$\frac{1}{{T_{{1_{i} }} }} + k_{{io}} ~(s^{{ - 1}} )$	R_11_	5.57 ± 1.07	5.80 ± 0.56	0.421	2.07 ± 1.09	2.17 ± 1.41	0.905
	*k*_oi_ (s^− 1^)	R_12_	3.06 ± 0.74	4.18 ± 0.46	0.310	4.18 ± 1.07	4.16 ± 2.56	0.841
	***k***_**io**_ **(s** ^**− 1**^ **)**	**R**_**21**_	**2.48** ± **0.24**	**2.34** ± **0.24**	**0.310**	**2.34** ± **0.40**	**1.99** ± **0.45**	**0.413**
	$\frac{1}{{T_{{1_{o} }} }} + k_{{oi}} ~(s^{{ - 1}} )$	R_22_	1.78 ± 0.26	1.74 ± 0.13	0.690	1.55 ± 0.72	1.29 ± 0.54	0.730
	Inward permeability (cm s^− 1^)		4.52 ± 0.87 × 10^− 5^	4.84 ± 0.42 × 10^− 5^	0.841	5.23 ± 2.03 × 10^− 5^	4.69 ± 2.79 × 10^− 5^	1.000
	Outward permeability (cm s^− 1^)		1.05 ± 0.10 × 10^− 4^	9.96 ± 1.02 × 10^− 5^	0.310	9.94 ± 1.71 × 10^− 5^	8.49 ± 1.95 × 10^− 5^	0.413
	$\frac{{\text{P}}_{\text{oi}}}{{\text{P}}_{\text{io}}}$		0.43 ± 0.06	0.49 ± 0.05	0.151	0.52 ± 0.16	0.53 ± 0.29	0.905

Bold values relate to the efflux rate constant, the most reliable parameter to estimate rate of cellular exchange and therefore subsequent membrane damage

When testing the hypothesis that differences would occur between the elements of the rate matrix of women with PE and that of healthy control pregnant women, it was found that no significant differences were observed for any element of the rate matrix, for either glucose anomer. We therefore conclude that the rate of carrier-mediated exchange of fluorinated glucose is the same for women suffering from PE as that of healthy pregnant women. In turn, the membrane of an erythrocyte from a woman suffering from PE is no more or less permeable to 3FDG than that of erythrocytes from healthy pregnant women.

When testing the hypothesis that no differences would be identified between the elements of the rate matrix from the 1D Selective Inversion and that of the 2D EXSY experiments, significant differences were observed for the sum of the longitudinal relation rate constant of the intracellular peak and efflux rate constant i.e. R_11_, $\frac{1}{{{T}_{{{1}_{i}}}}}+{{k}_{io}}$, (*p* = 0.008 for PE samples and 0.016 for control samples), and for both anomers of glucose (see Suppl Mat). Similarly, significant differences were found between the sum of the longitudinal relaxation rate constant of the extracellular peak and the influx rate constant (R_22_,$~\frac{1}{{{T}_{{{1}_{o}}}}}+{{k}_{oi}}$), determined using the 1D Selective Inversion, and that of the 2D EXSY, for the β-anomer only (*p* = 0.016). No other significant differences were observed between the results of the 1D and 2D experiments and the subsequently calculated permeabilities.

## Discussion

The NMR investigation into exchange across the erythrocyte membrane was successful in estimating the values of the rates of exchange of a mimic of a natural product, and has been useful in investigating the effect of preeclampsia on the intrinsic protein GLUT1 involved in this facilitated diffusion.

For comparison of rates of exchange and permeabilities of membranes, the efflux rate constant *k*_io_ and outward permeability P_io_ are the most reliable parameters (Kirk and Kuchel 1986; O’Connell et al. 1994; Kuchel et al. 1987). The efflux rate constant is independent of haematocrit. Once inside the cells, the rate at which a molecule leaves a cell will not depend on the total number of cells in the sample outside the membrane. Analogous to this, the outward permeability is calculated from the efflux rate constant, and is therefore not dependent on haematocrit. The mean efflux rate constants of 2.284 ± 0.695 and 2.200 ± 0.421 s^− 1^ for the α- and β-anomers of 3FDG respectively are comparable to those found previously in the literature as well as supporting anomeric preference for α-anomer (Kuchel et al. 1987; Potts and Kuchel 1992; London and Gabel 1995). This slight anomeric preference was explained by London and Gabel (1995), who showed that the α-anomer preferentially binds to the carrier on the inside of the membrane, due to the conformation of the carrier at that time inside the cell. After transportation, the conformation of this carrier changes outside the membrane, preferentially binding β-glucose. The higher rate obtained in this investigation into PE could be attributed to pregnancy in general as the permeability of erythrocytes may be affected by pregnancy. However it is not possible to confirm this without performing the same experiments on an equivalent number of non-pregnant controls.

No significant differences between the 1D Selective Inversion and the 2D EXSY for both the efflux rate constant and the outward permeability suggests that the results obtained are reliable and the methods robust. The only significant differences observed between the 1D and 2D data were in the elements of the rate matrix which are dependent on haematocrit. R_11_ includes the longitudinal relaxation rate of the broad intracellular peak $\frac{1}{{{T}_{{{1}_{i}}}}}$, whilst R_22_ includes the influx rate constant *k*_oi_ (see Suppl Mat). This difference can therefore be attributed to the estimation of peak volume and peak fitting in the 2D data due to the broadness of the intracellular peak, which is why previous studies favoured the 1D methods over 2D for simple two-site exchange (Perrin and Dwyer 1999; Engler et al. 1988; Robinson et al. 1985).

The substitution of a hydroxyl group for a fluorine atom on a glucose molecule does not seem to have an adverse effect on its exchange through the erythrocyte membrane. The exchange of 3FDG using the same protein as glucose has been demonstrated by Riley and Taylor (1973) who found that dilute solutions of glucose inhibited the transport of 3FDG. It has been suggested that the affinity of 3FDG for the binding site of the carrier is marginally higher, though not significantly so, than that of glucose itself, due to the F atom being directly involved in the hydrogen bonding in the binding site, mimicking that of the OH group of glucose (Riley and Taylor 1973; O’Connell et al. 1994). It is this hydrogen bonding which causes the difference in chemical shift between the intracellular and the extracellular populations. The intracellular hydrogen bonding will be different to that outside the cell as a result of the extent of the interactions present due to compartmentalisation and high protein concentration. The position of the fluorine atom on the hexose ring will also affect the extent of interactions. Preliminary investigations measuring the exchange of 2FDG clearly demonstrated this (see Suppl Mat). By increasing the osmolality of the wash solutions, the cellular volume is reduced, ensuring that the cytosol is isotonic with the extracellular medium, thus leading to a change in the intracellular interactions, and therefore a change in the chemical shift and broadness of the peak (Kirk and Kuchel 1985, 1988; Xu et al. 1991).

These effects, and the sharing of protein carrier GLUT1 by 3FDG and D-glucose, makes this study particularly useful in attempting to determine the effect of PE on the protein content of the erythrocyte membrane. Clearly, no significant differences between permeability or efflux rate constant of 3FDG between PE patients and healthy pregnant women showed that PE did not affect this protein part of the membrane. This result does not, however, rule out damage to the membrane by ROS of oxidative stress in PE, and does not contradict the results from earlier investigations; this study simply confirms that PE did not affect this particular protein transporter in executing facilitated diffusion of glucose and its mimics.

## Conclusions

^19^F-Dynamic NMR spectroscopy proved to be a successful technique in measuring the cellular exchange rate of analogues of endogenous metabolites—the results of both the 1D and 2D magnetisation transfer experiments suggest that preeclampsia does not have deleterious effects on the erythrocyte membrane protein involved in glucose exchange.

## Appendix

The 1D Selective Inversion experiment is performed over an array of mixing times, during which the labelled probe is transported across the cell membrane, allowing the nucleus of interest to precess at a different frequency to that at its previous location (Kuchel et al. 1988; Robinson et al. 1985; Gabel et al. 1997; O’Connell et al. 1994; London and Gabel 1995). The integrated intensities of the intracellular (I) and extracellular (E) peaks are measured for each mixing time throughout the range, whilst the intracellular peak is inverted. This procedure is repeated with the extracellular peak inverted. Additionally, it is necessary to ascertain the integrated intensities of both the I and E peak under equilibrium conditions (Bulliman et al. 1989; Perrin and Engler 1990). Once all integrated intensities have been obtained, matrices can be formed, based upon the Exchange Eq. (3):

3 $${{\bf{M}}_{\text{t}}}\text{ = }{{\text{e}}^{{-}\text{Rt}}}{{\bf{M}}_{\text{0}}}$$

where

4 $${\mathbf{M}}_{{\text{t}}} {\text{ = }}\left[ {\begin{array}{*{20}c} {{\text{A}}_{{{\text{ t}}_{{\text{m}}} }} } \ - {{\text{A}}_{{{\text{equi}}}} } \\ {{\text{B}}_{{{\text{ t}}_{{\text{m}}} }} } \ - {{\text{B}}_{{{\text{equi}}}} } \\ \end{array} } \right]\quad {\mathbf{M}}_{{\text{0}}} = \left[ \begin{gathered} {\text{A}}_{{{\text{t}}_{{\text{m}}} = 0}} - {\text{A}}_{{{\text{equi}}}} \hfill \\ {\text{B}}_{{{\text{t}}_{{\text{m}}} = 0}} - {\text{B}}_{{{\text{equi}}}} \hfill \\ \end{gathered} \right]$$

Matrices of (4) are calculated from three matrices produced directly from the integrated intensities. These three matrices, ${{\bf{M}}_{{{\text{t}}_{\text{m}}}}}$, ${{\bf{M}}_{{{\text{t}}_{\text{m}}}\text{=0}}}$ and ${{\bf{M}}_{\text{equilibrium}}}$ are initially formed in the same way as the Matrix in (5):

5 $${\bf{M}} = \left[ {\begin{array}{*{20}c} {{\text{A}}_{{{\text{A inverted}}}} } \ {{\text{A}}_{{{\text{B inverted}}}} } \\ {{\text{B}}_{{{\text{A inverted}}}} } \ {{\text{B}}_{{{\text{B inverted}}}} } \\ \end{array} } \right]$$

where, for example, A_A inverted_ is the integrated intensities of the resonance at site A, when this resonance is inverted. Matrices in (4) can be produced by simple subtraction. This process is repeated for each mixing time used in the range.

The Exchange Eq. (3) can then be linearised:

6 $${\mathbf{M}}_{{\text{t}}} = {\text{e}}^{{ - {\mathbf{R}}{\text{t}}}} {\mathbf{M}}_{{\text{0}}} \to {\text{ln}}\left( {{\mathbf{M}}_{{\text{t}}} {\mathbf{M}}_{{\text{0}}} ^{{ - {\text{1}}}} } \right) = - {\mathbf{R}}{\text{t}}$$

However, the difficulty of calculating the logarithm of a matrix is circumvented by using an alternative solution (7) where exponentials are eliminated:

7 $${\text{ln}}\left( {{\mathbf{M}}_{{\text{t}}} {\mathbf{M}}_{{\text{0}}} ^{{ - {\text{1}}}} } \right) = {\mathbf{X}}\left( {\ln \Lambda } \right){\mathbf{X}}^{{ - 1}} = - {\mathbf{R}}{\text{t }}$$

Here **X** is the square matrix of eigenvectors of (M_t_M_0_^−1^), X^− 1^ is its inverse, and ln Λ is the diagonal eigenvalue matrix (Jeener et al. 1979; Bremer et al. 1984; Johnston et al. 1986; Hernandez-Garcia et al. 2007; Szekely et al. 2006). This is formed as ln Λ = diag(ln λ), as shown in the Matrix of [8] (Johnston et al. 1986):

8 $$\ln \Lambda = \left[ {\begin{array}{*{20}{c}} {\ln {\lambda _1}}&\ 0\\ 0&\ {\ln {\lambda _2}} \end{array}} \right]$$

These linearised data can then be plotted as a function of mixing time, as shown in Fig. 5. The gradients of the best-fit straight lines produced will give the elements of the square rate matrix R (Johnston et al. 1986; Engler et al. 1988; O’Connell et al. 1994). In this case of two-site exchange across the erythrocyte membrane, these elements will form the 2 × 2 rate matrix R (Potts and Kuchel 1992; Gabel et al. 1997; Bulliman et al. 1989; Szekely et al. 2006) Eq. (9):

9 $$\boldsymbol{R} = \left[ {\begin{array}{*{20}{c}} {\frac{1}{{{T_{{1_A}}}}} + {k_A}}&{ - {k_B}}\\ { - {k_A}}&{\frac{1}{{{T_{{1_B}}}}} + {k_B}} \end{array}} \right]$$

The linear equations of the lines with negative gradient will correspond to the first-order influx and efflux rate constants, whilst those with positive slope give the sum of the longitudinal relaxation rate constants of the I and E peaks and the exchange rate constants (Bulliman et al. 1989; Perrin and Engler 1990).

The same principles apply to the analysis of the 2D EXSY data as that of the 1D Selective Inversion although the application is slightly different. Whilst a range of mixing times is still required, the longer acquisition time of the 2D EXSY imposes some restrictions on the number of mixing times used and therefore it is usual to use fewer mixing times than with the 1D equivalent. A 2D EXSY experiment is performed for each mixing time; one of these mixing times used must be 0 (Johnston et al. 1986; O’Connell et al. 1994; Gabel et al. 1997; Perrin and Dwyer 1999).

The matrix methods differ in that the matrices M_t_ $\left( {{{\bf{M}}_{{{\text{t}}_{\text{m}}}}} - {{\bf{M}}_{{\text{equi}}}}} \right)$ and M_0_ $\left( {{{\bf{M}}_{{{\text{t}}_{\text{m}}} = 0}} - {{\bf{M}}_{{\text{equi}}}}} \right)$ of [4] are produced directly from the volumes of cross peaks and diagonal peaks of the of the spectra at each mixing time (M_t_), including when *t*_m_ = 0 (M_0_) (O’Connell et al. 1994; Johnston et al. 1986).

If:

10 $$\left( {{{\bf{M}}_{\text{t}}}{{\bf{M}}_{\text{0}}}^{{{ - 1}}}} \right) = {\bf{A}} = {{\text{e}}^{ - {\bf{R}}{\text{t}}}}$$

then:

11 $$- {\bf{R}} = \frac{1}{{{{\text{t}}_{\text{m}}}}}\ln {\bf{A}}$$

or, from (7):

12 $$- {\bf{R}} = \frac{1}{{{{\text{t}}_{\text{m}}}}}{\bf{X}}\left( {\ln {\varvec{\Lambda }}} \right){{\bf{X}}^{ - 1}}$$

where X is the square matrix of eigenvectors of A, X^− 1^ is its inverse, and ln Λ is the diagonal eigenvalue matrix (Jeener et al. 1979; Johnston et al. 1986; Macura and Ernst 1980). Clearly, this procedure is identical to that of the 1D Selective Inversion analysis, but with the alternative direct formation of matrix A from the 2D NMR data (Johnston et al. 1986):

13 $${\mathbf{A}} = \left[ {\begin{array}{*{20}c} {\frac{{a_{{AA}} }}{{A_{0} }}} & {\frac{{a_{{AB}} }}{{B_{0} }}} \\ {\frac{{a_{{BA}} }}{{A_{0} }}} & {\frac{{a_{{BB}} }}{{B_{0} }}} \\ \end{array} } \right]$$

where *a*_*AA*_ and *a*_*BB*_ are the diagonal peak amplitudes of site A and site B in an experiment with mixing; *a*_*AB*_ and *a*_*BA*_ are the cross peak amplitudes (showing exchange between site A and site B) in an experiment with mixing; and *A*_0_ and *B*_0_ are the diagonal peak amplitudes of site A and site B in an experiment without mixing (*t*_m_ = 0).

## Electronic supplementary material

Below is the link to the electronic supplementary material.

Supplementary material 1 (PDF 589 KB)
