# Supplementary material for: Determination of glucose exchange rates and permeability of erythrocyte membrane in preeclampsia and subsequent oxidative stress-related protein damage using dynamic-19F-NMR
Source: J Biomol NMR. 2017 Feb 21;67(2):145–56. doi: 10.1007/s10858-017-0092-y (PMC5346149; doi:10.1007/s10858-017-0092-y)

Determination of glucose exchange rates and permeability of erythrocyte membrane in preeclampsia and subsequent oxidative stress-related protein damage using dynamic-^{19}F-NMR

Elizabeth Dickinson[a*], John R. P. Arnold[b], and Julie Fisher[c]

[a] Department of Chemistry, University of York, Heslington, York, UK.
elizabeth.dickinson@york.ac.uk
Tel: (+44) (0)1904 322673
Fax: (+44) (0)1904 323433
[b] Selby College, Abbot's Road, Selby, North Yorkshire, YO8 8AT, UK.
[c] School of Chemistry, University of Leeds, Leeds, UK **(IN MEMORIAM)**

Table 2 The comparison of elements of the rate matrix obtained from the 1D and 2D experiments.

Anomer	Element of Rate Matrix And Permeabilities		*p-value*	
			PE	CONTROL
β	$\frac{1}{T_{1_i}} + k_{io}$ (s^{-1})	R_{11}	**0.008**	**0.016**
	k_{oi} (s^{-1})	R_{12}	0.548	1.000
	k_{io} (s^{-1})	R_{21}	0.310	0.905
	$\frac{1}{T_{1_o}} + k_{oi}$ (s^{-1})	R_{22}	0.690	**0.016**
	Inward Permeability (cm s^{-1})		0.421	0.413
	Outward Permeability (cm s^{-1})		0.310	0.905
	$\frac{P_{oi}}{P_{io}}$		1.000	0.703
α	$\frac{1}{T_{1_i}} + k_{io}$ (s^{-1})	R_{11}	**0.008**	**0.016**
	k_{oi} (s^{-1})	R_{12}	0.841	0.690
	k_{io} (s^{-1})	R_{21}	0.841	0.190
	$\frac{1}{T_{1_o}} + k_{oi}$ (s^{-1})	R_{22}	0.421	0.286
	Inward Permeability (cm s^{-1})		0.690	0.730
	Outward Permeability (cm s^{-1})		0.841	0.190
	$\frac{P_{oi}}{P_{io}}$		0.310	0.286

Figure 6: (Top) ^{19}F-NMR spectrum and structure of the anomers of 2FDG in D_2O, at 470.34 MHz and at 37°C; (Centre and Bottom) spectra of erythrocytes washed with 2FDG solution (B), buffered with Tris-HEPES, then broadband proton decoupled (A).

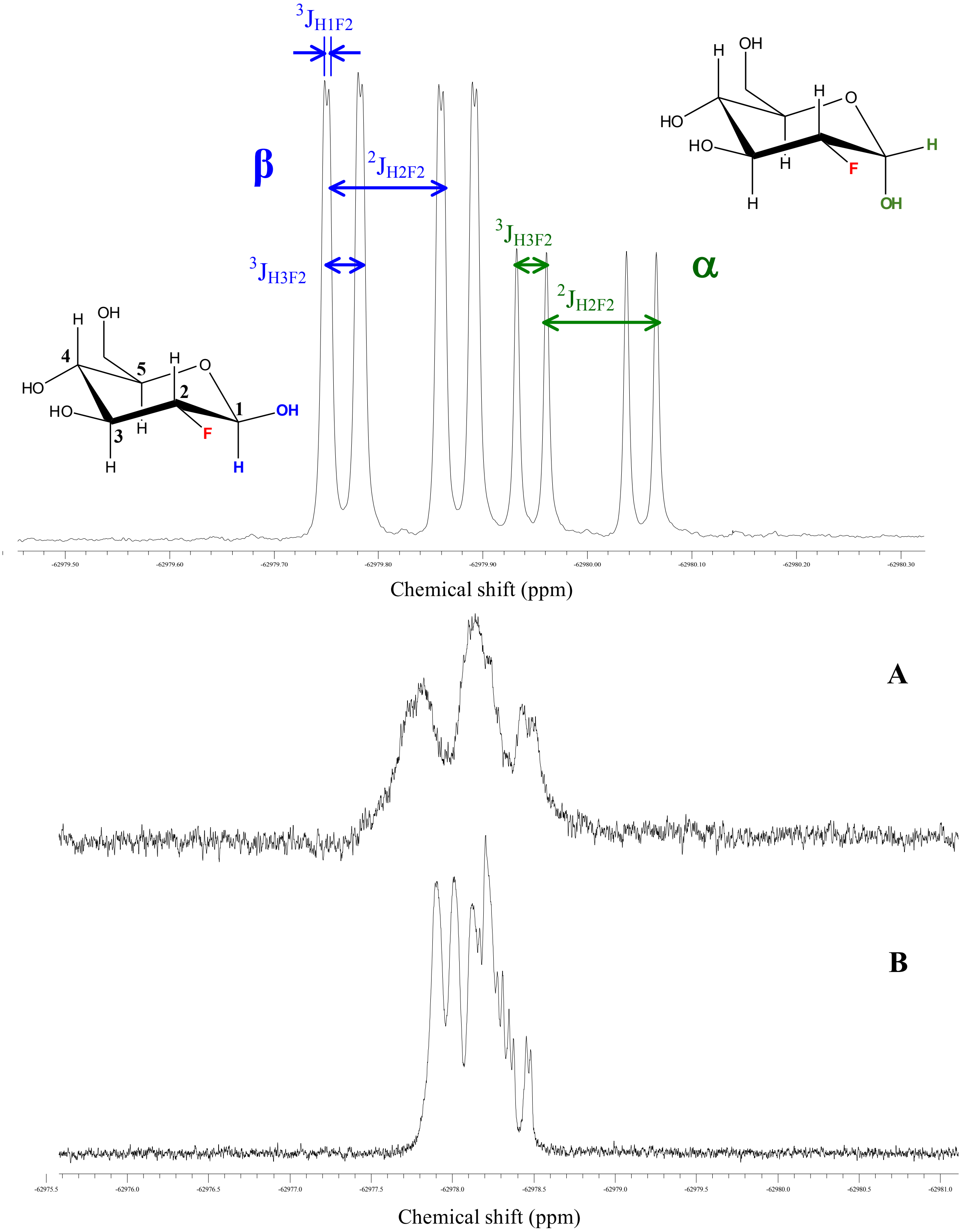

Figure 7: Broadband proton decoupled ^{19}F NMR spectra of erythrocytes washed with 2FDG solution, buffered with PBS at (Top) 40 mM concentration and (Bottom) 200 mM concentration.

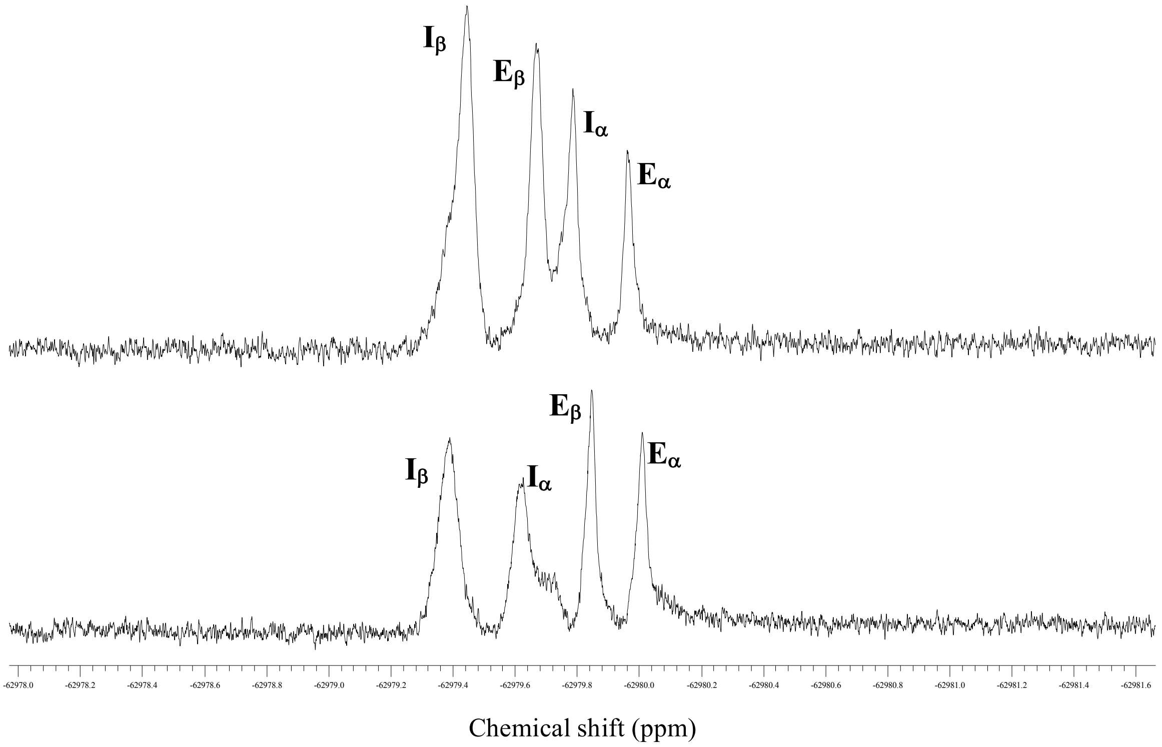

Supplement: Supplementary file 1 — Supplementary material 1 (PDF 589 KB) [file 10858_2017_92_MOESM1_ESM.pdf]
